# Basic and clinical immunology – 3025. Suppression of eosinphil activation by levocetirizine hydrochloride *in vivo*

**DOI:** 10.1186/1939-4551-6-S1-P201

**Published:** 2013-04-23

**Authors:** Kenichiro Kawaguchi, Kenichi Kanai, Kazuhito Asano, Takeyuki Sanbe, Harumi Suzaki

**Affiliations:** 1 Otorhinolaryngology, Showa University, Tokyo, Japan; 2 Physiology, Showa University, Japan

## Background

Histamine H_1_ receptor antagonists are used for the treatment of allergic disorders such as allergic rhinitis and atopic allergy with remarkable success. However, the influence of antihistamines on the function of eosinophils, which are the most important final effector cells in allergic diseases, is not well understood.

## Methods

The influence of histamine H_1_ receptor antagonists on eosinophil functions was examined through the choice of levocetirizine hydrochloride (LH) *in vivo*.Patients with Japanese cedar pollinosis were orally treated with LH once a day at a single dose of 5 mg for two weeks during Japanese cedar pollen season (February 2012 to April 2012). Nasal secretions were obtained before and after treatment with the filter paper method. Eosinophil activation was assessed by measuring the levels of both ECP and MBP in nasal secretions by ELISA. We also examined the number of eosinophils in nasal secretions and IgE levels in peripheral blood obtained from patients before and after treatment with LH.

## Results

Oral administration of LH could not suppress both peripheral blood eosinophila and IgE hyper-production. On the other hand, ECP and MBP levels in nasal secretions decreased significantly after treatment with LH. LH treatment also favorably modified the clinical conditions of patients: the clinical symptom scores, such as sneezing, nasal discharge and congestion decreased significantly after treatment with LH.

## Conclusions

These results may suggest that LH exerts inhibitory effects on eosinophil activation and results in favorable modification of clinical status of pollinosis patients.

